# The Human Impact of Tsunamis: a Historical Review of Events 1900-2009 and Systematic Literature Review

**DOI:** 10.1371/currents.dis.40f3c5cf61110a0fef2f9a25908cd795

**Published:** 2013-04-16

**Authors:** Shannon Doocy, Amy Daniels, Anna Dick, Thomas D. Kirsch

**Affiliations:** Johns Hopkins Bloomberg School of Public Health, Baltimore, Maryland, United States; Johns Hopkins Bloomberg School of Public Health, Baltimore, Maryland, United States; Johns Hopkins Bloomberg School of Public Health, Baltimore, Maryland, United States; Johns Hopkins University School of Medicine and Bloomberg School of Public Health, Baltimore, Maryland, United States

## Abstract

Introduction. Although rare, tsunamis have the potential to cause considerable loss of life and injury as well as widespread damage to the natural and built environments. The objectives of this review were to describe the impact of tsunamis on human populations in terms of mortality, injury, and displacement and, to the extent possible, identify risk factors associated with these outcomes. This is one of five reviews on the human impact of natural disasters. Methods. Data on the impact of tsunamis were compiled using two methods, a historical review from 1900 to mid 2009 of tsunami events from multiple databases and a systematic literature review to October 2012 of publications. Analysis included descriptive statistics and bivariate tests for associations between tsunami mortality and characteristics using STATA 11. Findings. There were 255,195 deaths (range 252,619-275,784) and 48,462 injuries (range 45,466-51,457) as a result of tsunamis from 1900 to 2009. The majority of deaths (89%) and injuries reported during this time period were attributed to a single event –the 2004 Indian Ocean tsunami. Findings from the systematic literature review indicate that the primary cause of tsunami-related mortality is drowning, and that females, children and the elderly are at increased mortality risk. The few studies that reported on tsunami-related injury suggest that males and young adults are at increased injury-risk. Conclusions. Early warning systems may help mitigate tsunami-related loss of life.

## Introduction

A tsunami, the Japanese word for “large harbor wave,” is a series of large water waves produced by a sudden vertical displacement of water. Aquatic earthquakes are the most common cause, but volcanic activity, landslides and impacts of meteorites may also generate tsunamis. Earthquake-generated tsunamis develop when tectonic plates, either deep sea, continental shelf, or coastal, move abruptly in a vertical direction, and the overlying water is displaced. Waves created by these disturbances move in an outward direction, away from the source. In deep waters, the surface disturbance of water is relatively unnoticeable and may only be felt as a gentle wave. As the wave approaches shallow waters along the coast, it rises above the surface related to the amplitude of the underwater waves. The speed of the tsunami diminishes and the height of the wave increases as it reaches the shore line. The extent of inundation that occurs is largely dependent on local topography; in low lying areas flooding can be extensive and can reach far inland disrupting even non-coastal communities 1 ^,^ 2 ^,^ 3 .

While rare, high-impact tsunamis have the potential to cause widespread destruction and affect hundreds of thousands 4 ^,^ 5 . The 2004 Indian Ocean tsunami resulted in more than 225,000 deaths across twelve nations, and the 2011 Japan tsunami caused an estimated 28,000 deaths 6. Displacement and damage to infrastructure are also important contributors to the human, social, and economic effects of tsunamis 7. Few reviews of the impacts of tsunamis on human populations exist. Given the recent tsunami disasters in 2004 and 2009, a broader understanding of the characteristic effects of tsunamis on human populations could inform preparedness and response efforts. The objectives of this review were to describe the impact of tsunamis on the human population, in terms of mortality, injury, and displacement and to identify risk factors associated with these outcomes. This is one of five reviews on the human impact of natural disasters, the others being volcanoes, floods, cyclones, and earthquakes.

## Methods

Data on the impact of tsunamis were compiled using two methods, a historical review of tsunami events and a systematic literature review for publications relating to the human impacts of tsunamis with a focus on mortality, injury, and displacement.

**Historical Event Review**

Data for the historical event review were obtained from two sources. The National Oceanic and Atmospheric Administration’s National Geophysical Data Center (NOAA-NGDC) tsunami database 8 consists of two sets of related files on tsunami events and a tsunami run-ups. The event file lists the cause (almost exclusively undersea earthquakes) that triggered tsunamis together with the total impact of a single event (i.e. the aggregate impact from multiple wave run-up locations together with the coordinates of the originating event). The run-up file includes wave characteristics and impacts in each affected location (multiple run-up reports per event). Records from the tsunami run-up file that met all of the following criteria were retained: 1) occurred between 1900 and 2009; 2) reported as definitely or probably occurring; 3) had a wave height ≥2.0m; and 4) resulted in ≥1 deaths. This yielded a total of 116 run-up reports from 82 different tsunami events.

The Centre for Research on the Epidemiology of Disasters’ Emergency Events (CRED EM-DAT) was the second data source used in the review. All wave/surge events that were reported between 1900 and 2009 in EM-DAT were included (n=58); data were initially exported in 2008 when CRED reported wave/surge events; this category was subsequently discontinued. For tsunami impacts reported by EM-DAT, zeroes were treated as missing values because they were used as placeholders and their inclusion in the analysis could contribute to the under estimation of tsunami impacts. The NOAA-NGDC run-up database was subsequently searched for events reported by EM-DAT that were previously excluded due to wave height <2.0m or uncertain reporting criteria, and NOAA-NGDC event data were added to the EM-DAT records. This process yielded a total of 151 records, including 58 events reported by EM-DAT and 134 run-ups reported by NOAA-NGDC. The run-up file was used to assess wave characteristics and outcomes. A separate event file comprised of 81 events was created by combining multiple reports of tsunami impact within a country into a single event. To create a summary record for each of event with multiple reports, human impacts at each location were summed, and the maximum wave height and inundation depth were applied. The event file had 94 events, including 58 reported by EM-DAT and 71 by NOAA, and was used to assess frequency and distribution of tsunamis and their impact by country. Findings presented in this review are based on the 151 run up file. Both run-up and source data can be accessed online at http://www.jhsph.edu/refugee/natural_disasters/_Event_Tsunamis.html.

In order to examine country- and event-specific characteristics associated with low and high levels of tsunami mortality, deaths were categorized as follows: low (<10 deaths), medium (11-75 deaths) and high (>75 deaths). Bivariate tests for associations between tsunami mortality and the following characteristics were performed using χ^2^ (categorical measures) and ANOVA (continuous measures): time period (dichotomized, 1900-1955 and 1956-2009), region as defined by the World Health Organization (WHO), income level (World Bank), gross domestic product (GDP), GINI (measure of income inequality), distance from source (quartiles), wave height (dichotomized, <6.65 and >6.65) and earthquake magnitude. For the region variable, only three events were reported in Africa and because all were related to the 2004 Indian Ocean tsunami, they were grouped with Southeast Asian; only one event was recorded in the Eastern Mediterranean region which was grouped Europe .All analyses were performed using Stata Statistical Software, Version 11.0 9

***Systematic Literature Review***

Key word searches in MEDLINE (Ovid Technologies, humans), EMBASE (Elsevier, B.V., humans), SCOPUS (Elsevier B.V., humans), and Web of Knowledge, Web of Science (Thomson Reuters) were performed to identify articles published in July 2007 or earlier that described natural hazards and their impact on human populations. Key words used to search for natural hazards included *natural hazard(s), natural disaster(s), volcano(es), volcanic, volcanic eruption, seismic event, earthquake(s), cyclone(s), typhoon(s), hurricane(s), tropical storm(s), flood(s), flooding, mudslide(s), tsunami(s), and tidal wave(s).* Key words included for impact on human populations were *affected, damage(d), injury, injuries, injured, displaced, displacement, refugees, homeless, wounded, wound(s), death(s), mortality, casualty, casualties, killed, died, fatality, fatalities* and had to be used in either the title, abstract or as a subject heading/key word. The search resulted in 2,747 articles from MEDLINE, 3,763 articles from EMBASE, 5,219 articles from SCOPUS, and 2,285 articles from ISI Web of Knowledge. Results from the four databases were combined and duplicates were excluded to yield a total of 9,958 articles. . One search was done for all the five natural hazards described in this set of papers. This paper describes the results for tsunamis. The systematic review is reported according to the PRISMA guidelines.

Title screening was performed to identify articles that were unrelated to natural disasters or human populations. Each title was screened by two independent reviewers and was retained if either or both reviewers established that inclusion criteria were met. To ensure consistent interpretation of inclusion criteria, percent agreement was assessed across reviewers for a small sample of articles, and title screening began after 80% agreement on inclusion was achieved. A total of 4,873 articles were retained for abstract review. Articles that met one or more of the following criteria were excluded in the abstract screening: language other than English; editorial or opinion letter without research-based findings; related to environmental vulnerability or hazard impact but not human populations; individual case report/study; focus on impact/perceptions of responders; and not related to human or environmental vulnerabilities or impacts of hazards. As with the title screening, overall percent agreement between reviewers was assessed, and abstract screening began after achieving 80% agreement. Each abstract was screened by two independent reviewers and was retained if either or both reviewers established that inclusion criteria were met. During the abstract review, included abstracts were coded for event type, timeframe, region, subject of focus, and vulnerable population focus.

A total of 126 articles were retained for full article review. Articles discussing the impacts of natural disasters on human populations in terms of mortality, injury, and displacement were prioritized for review. A total of 64 articles on tsunamis meeting the aforementioned subject focus criteria were retained for full review. Upon full review, 27 articles were retained including 23 that underwent dual review and standardized data abstraction, two identified as review articles 10 ^,^ 11, one policy article 12 and one article on mitigation 13 (Figure 1). Following the systematic review, a hand search was conducted using the databases and key words listed above to identify relevant articles published between July 2007 when the initial search was conducted and October 2012; seven additional articles were identified that met criteria for full review. In total, 34 articles relating to risk factors for mortality, injury or displacement were identified; summaries of articles with primary data (n=30) are presented in Table 1.

**Overview of the systematic literature review process for tsunamis:**
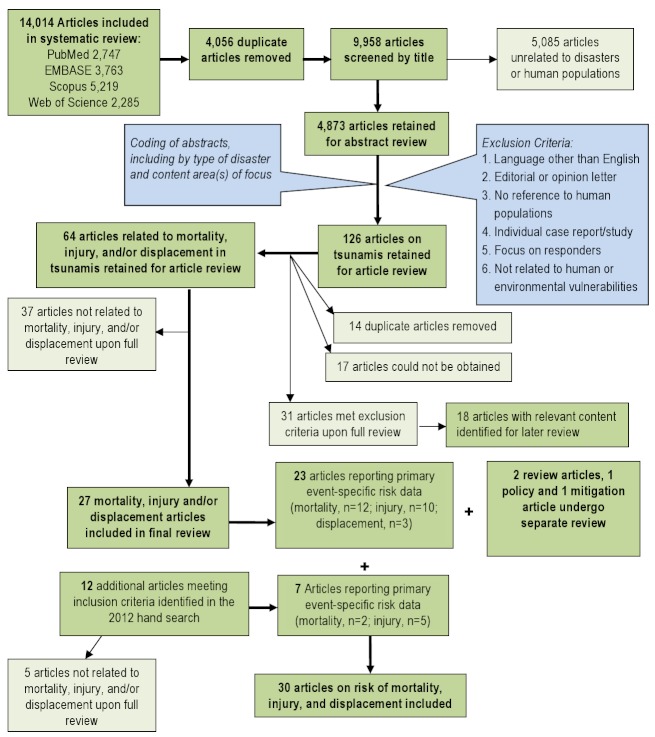

**Table 1: Articles included in the tsunami systematic literature review relating to mortality and injury* (N=30):** * Displacement is excluded from the table because no primary data on displacement was collected in only three studies: MMWR, 2006; Rofi, 2006; and Yamada, 2006; ** Additional articles from the hand search through October 2012

**Article**	**Event**	**Summary**	**Mortality (n=14)**	**Injury & Morbidity (n=19)**
Tsuji et al., 1995^15^	June 3, 1994, East Java, Indonesia	Field survey to assess the destruction of the tsunami	223 deaths reported	Neither reported
Tsuji et al., 1995^16^	December 12, 1992, Flores Island	Field survey to assess the destruction of the tsunami	1690 deaths reported	Neither reported
Davies et al., 2003^17^	July 17, 1998, Aitape, Papua New Guinea , 1998	Interviews and field investigations to describe the physical characteristics of tsunamis.	1600 deaths reported; primary causes of death were drowning and impacts with hard objects	Neither reported
Brennan and Rimba., 2005^18^	December 26, 2004, Indonesia	Rapid health assessment to determine the public health impact of the tsunami in three communities of the Aceh Jaya district	70% of the population died	Injury data not reported85% of children <5 yrs old experienced an illness
Calder & Mannion, 2005^19^	December 26, 2004, Sri Lanka	Review of findings from a DFID needs assessment with an emphasis on trauma/ orthopedic and psychiatric services	Not reported	100 injuries reportedNo morbidity data reported
Johnson & Travis, 2005^20^	December 26, 2004, Thailand	Facility-based, retrospective record review to describe tsunami-related injuries at the provincial hospital in Krabi province.	25 deaths reported	1357 injuries reportedNo morbidity data reported
Lee et al., 2005^21^	December 26, 2004, Indonesia	Description of primary health care services delivered in an internally displaced persons camp by a medical team from Singapore	Not reported	1958 people injuredNo morbidity data reported
Lim et al., 2005^22^	December 26, 2004, Sri Lanka	A description of the patients treated by two Korean medical teams over a nine day period following the tsunami	Not reported	4710 injuries reported; primary causes were running from the tsunami and surviving in wreckageNo morbidity data reported
Maegele et al., 2005^23^	December 26, 2004, Thailand	Observational study of patients seen at an adult intensive care unit a university hospital	Not reported	17 injuries reported; the primary cause of injury was due to hitting floating debrisNo morbidity data reported
Chambers et al., 2006^24^	December 26, 2004, Indonesia	Description of surgical and humanitarian assistance operations of a joint Australian and New Zealand operation in the four week period following the tsunami	Not reported	71 injuries reportedNo morbidity data reported
Fan, 2006^25^	December 26, 2004, Indonesia	Description of patients treated in Banda Aceh by a medical team from Singapore in the first few weeks following the Tsunami	Not reported	2183 injuries reported; primary causes was being caught in the wave and struck by debris. No morbidity data reported
Kwak et al., 2006^26^	December 26, 2004, Sri Lanka	Descriptive study of patients treated by Korean surgical and medical personnel from January 2 to 8, 2005	Not reported	2807 individuals treated for medical problems (82%) and injuries (18%)
MMWR, 2006^27^	December 26, 2004, Indonesia	Three household surveys to assess affected populations and evaluate effectiveness of relief interventions 7 months post-disaster	Not reported	Neither reported
Nishikiori et al., 2006^28^	December 26, 2004, Sri Lanka	Household survey to assess mortality among the internally displaced population	446 deaths reported	Neither reported
Nishikiori et al., 2006^29^	December 26, 2004, Sri Lanka	Household survey to assess mortality among the internally displaced population	446 deaths reported; primary cause of death was drowning	Neither reported
Redwood –Campbell & Riddez, 2006^30^	December 26, 2004, Indonesia	Descriptive study of outpatients at an International Committee of the Red Cross hospital nine weeks following the tsunami	Not reported	271 injuries reported
Rodriguez et al., 2006^31^	December 26, 2004, India and Sri Lanka	Observations and key-informant interviews to describe the societal impacts of the disaster	250000 deaths reported; primary cause for death was drowning	Neither reported
Rofi, et al., 2006^32^	December 26, 2004, Indonesia	Household survey to estimate mortality and displacement	295 deaths reported	Neither reported
Roy, 2006^33^	December 26, 2004, India	Descriptive study of deaths and individuals treated at a secondary care hospital in the days following the tsunami	62 deaths reported; primary cause of death was drowning	Minor injuries reported 17% of patients showed symptoms of PTSD
van Griensven et al., 2006^34^	December 26, 2004, Thailand	Multi-stage, cluster survey to assess the mental health of displaced and non-displaced populations following the tsunami	Not reported	Injury data not reported1061 mental health issues reported; primary cause due to tsunami
Yamada et al., 2006^35^	December 26, 2004, Sri Lanka	Needs assessment conducted to understand tsunami impact on specific population groups and on the health care system	Not reported	Injury data not reportedGeneral mental health consequences of the disaster reported
Doocy et al., 2007^36^	December 26, 2004, Indonesia	Estimation of tsunami mortality using GIS-based vulnerability modeling	131066 deaths estimated	Neither reported
Doocy et al., 2007^37^	December 26, 2004, Indonesia	Four two-stage cluster household surveys to assess mortality and associated risk factors	1642 deaths reported	Neither reported
Johnson & Travis, 2006^38^**	December 26, 2004, Thailand	A description of individuals treated at a provincial tertiary hospital in the weeklong period following the event	Not reported	1357 injuries reported
Johnson & Travis, 2006^39^**	December 26, 2004, Thailand	Application of the tri-modal death model to mortality and injury post-tsunami	Not reported	Not reported
Meynard et al., 2008^40^**	December 26, 2004, Indonesia	Cluster survey s to assess health of children affected by the disaster	Not reported	Injury data not reported7-13% malnourished and 68% experienced sickness
Prasartritha et al., 2008^41^**	December 26, 2004, Thailand	Retrospective record review of injury care seekers at three hospitals	Not reported	2311 injuries reportedMorbidity data not reported
Doocy et al., 2009^42^**	December 26, 2004, Indonesia	Three two-stage cluster household surveys to assess injury and associated risk factors	17.7% (CI:16.8-18.6) of the population died	707 injured individualsMorbidity data not reported
Doung-ngern et al., 2009^43^**	December 26, 2004, Indonesia	Assessment of wound treatment among care seekers at four public hospitals	Not reported	513 injuries (wounds) reportedMorbidity data not reported
Nagamatsu et al, 2012^44^**	March 11, 2011 Japan	Review of DMAT response	282 deaths from deteriorating pre-existing chronic medical conditions	4891 injured patients at the Ishinomaki Red Cross Hospital

## Results

**Historical Event Review**

Between 1900 and 2009, 94 tsunamis that affected human populations were recorded. The frequency of events was relatively constant through the 1980s, after which a dramatic increase was reported (Figure 2). This increase is likely the result of improvements to monitoring and reporting systems. Tsunami frequency and mortality were concentrated in the Western Pacific, Southeast Asia, and Americas regions, each of which accounted for almost one third of tsunami events and deaths, but Southeast Asia accounted for 52% of the tsunami-affected population from 1900 – 2009 and 95% of the tsunami affected population from 1980 - 2009 (Figure 4). An estimated 2.5 million people were affected by tsunamis between 1900 and 2009. A sharp increase in tsunami mortality and affected populations was observed from 2000 to 2009 as a result of the 2004 Indian Ocean tsunami (Figure 3). The overall impact of tsunamis on human populations is summarized in Table 2.

**Reporting of tsunamis by source and decade:**
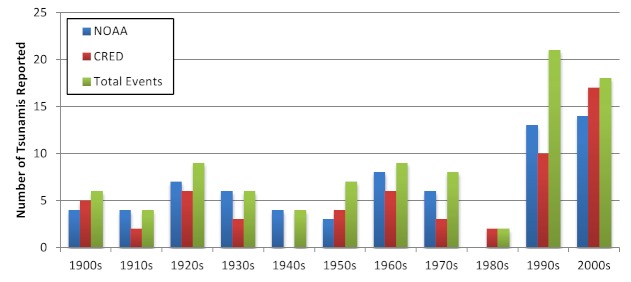

**Tsunami events affecting human populations by decade:**
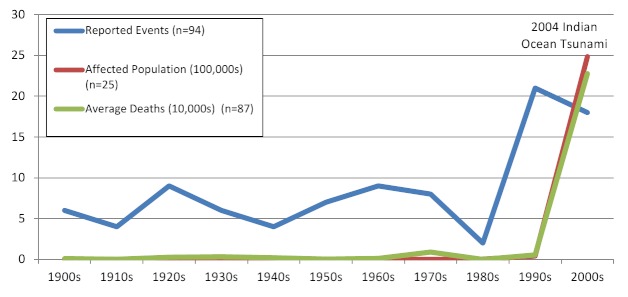

**Tsunamis and their impact on human populations by region, 1980-2009*:**
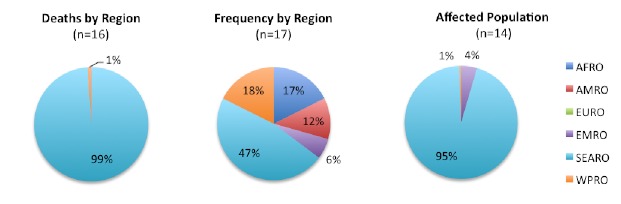
*Regions as defined by the World Health Organization

**Table 2: Summary measures for impact of tsunamis on human populations, 1900-2009 (n=94) and 1980-2009 (n=16):** Notes: figures based on the highest reported number of deaths or injuries in an event in one country. Homeless and total affected populations are reported only by EM-DAT, thus ranges are not presented for overall impact estimates.

***Cumulative Impact of Tsunamis, 1900-2009 [1970-2009]***								
***Human Consequence***	***# of Events***		***Best Estimate***		***Range***			
	**1900-2009 (n=94)**	**1980-2009 (n=16)**	**1900-2009 (n=94)**	**1980-2009 (n=16)**	**1900-2009 ** **(n=94)**		**1980-2009** **(n=16)**	
*Deaths*	87	16	*255,195*	*230,012*	*252,619-275,784 *		228,932 - 231,091	
*Injuries*	22	9	*48,462*	*14,029*	*45,466-51,457 *		11,033 - 17,024	
*Homeless*	12	7	*1,081,764*	*1,034,214*	*---*		---	
*Total Affected*	25	4	*2,532,189*	*2,479,689*	*---*		---	
Event Summary Statistics								
***Human Consequence***	***# of Events***		***Median***		***Mean***		***Range***	
	**1900-2009 (n=94)**	**1980-2009 (n=16)**	**1900 - 2009 (n=94)**	**1980-2009 (n=16)**	**1900-2009 (n=94)**	**1980-2009 (n=16)**	**1900-2009 (n=94)**	**1980-2009 (n=16)**
***Events with deaths***	87 [92.5%]	16 [100%]	50		2,963	14,839	1-165,708	1-165,708
Reported by EM-DAT	53 [56.4%]	16 [100%]	64	91	4,559	14,339	1-165,708	1-165,708
Reported by NOAA	69 [73.4%]	15 [93.75%]	61	108	3,623	15,375	1-165,659	1-165,659
***Events with Injuries***	22 [23.4%]	9 [56.25%]	218	2,878	2,339	5,255	2-23,176	2-23,176
EM-DAT	13 [13.8%]	9 [56.25%]	543	2,214	3,320	5,113	2-23,176	2-23,176
NOAA	10 [10.5%]	10 [6.25%]	127	6,534	885	6,534	7-6,534	---
**Homeless, all events**	12 [12.8%]	12 [43.75%]	4773	4,296	90,147	147,745	70-532,898	70-532,898
**Total Affected, all events**	25 [26.6%]	25 [87.5%]	5063	21,457	101,288	177,745	2-1,109,306	194-1,109,306

*Tsunami Characteristics.* The physical characteristics of tsunamis were reported by NOAA-NGDC and included distance of the tsunami run-up from source, wave height, and earthquake magnitude. The median distance from source was 119 km (mean 810 km; range 7-10,621 km), and the median wave height was 6.7m (mean 13.0m; range 1.8 -67.1m). The majority of the tsunamis reported were due to earthquakes (95.5%), with small minorities resulting from landslides (3.0%), volcanoes (0.8%), and meteorological events (0.8%). Median magnitude for earthquake generated tsunamis was 8.1 (mean 8.1; range 6.3-9.5).

*Mortality.* Deaths were reported in 92.6% (n=94) of tsunamis occurring between 1900 and 2009. There were an estimated 255,195 deaths (range 252,619-275,784) resulting from tsunamis recorded in the historic event review, with the 2004 Indian Ocean tsunami accounting for an estimated 227,497 deaths (89%) of all mortality. Deaths were concentrated in Indonesia (62% or 170,689 deaths), Sri Lanka (13% or 35,399 deaths), Thailand (3.2%, 8,876 deaths) and the Philippines (3% or 8,137 deaths). Apart from rare high-impact events such as the 2004 Indian Ocean tsunami, mortality levels in tsunamis tend to be relatively low with a median of 50 deaths per event (mean=2,963,range 0-165,708) when using the highest reported death toll.

Table 3 presents results of the bivariate analyses between tsunami characteristics and mortality. Time period, and GINI coefficient were not statistically associated with tsunami mortality. There were considerable differences in mortality levels by WHO region, with the majority of tsunami events that occurred in the Americas resulting in low (<10) deaths and 50% of events in the South East Asian region resulting in high (>75) deaths (*p*<.001). Both World Bank-defined income level and per capita GDP were significantly associated with tsunami mortality. Whereas the majority of events in high income countries resulted in ten or fewer deaths, a considerably greater number of events in low and lower-middle income countries resulted in greater than ten deaths (*p*=.009). Similarly, events that resulted in greater than 75 deaths occurred in countries or territories with per capita GDPs that were more than two times lower than countries that experienced fewer tsunami deaths (*p*<.001). While the data show an inverse associations between tsunami mortality and distance from source (*p*=.001) and a positive association between mortality and wave height (*p*=.016), it is important to note that information pertaining to both measures were missing in a considerable proportion of events, particularly those occurring earlier in the study period.

**Table 3: Bivariate associations between country and event characteristics and tsunami mortality:** 

Characteristic	<=10 deaths (n = 57)	11-75 deaths (n = 51)	>75 deaths (n = 44)	P-value
**Time period, n (%)**				
1900-1955	26 (41)	22 (35)	15 (24)	.485
1956-2009	31 (35)	29 (33)	29 (33)	
**WHO Region, n (%)**				
Americas	36 (63)	12 (21)	9 (16)	<.001
Western Pacific	8 (21)	19 (50)	11 (29)	
Southeast Asia & Africa	8 (20)	12 (30)	20 (50)	
Europe & Eastern Mediterranean	5 (29)	8 (47)	4 (24)	
**World Bank Development Level, n (%)**				
Low income	6 (38)	6 (38)	4 (25)	.009
Lower-middle income	10 (20)	17 (35)	22 (45)	
Upper-middle income	4 (25)	6 (38)	6 (38)	
High Income	37 (52)	22 (31)	12 (17)	
**Distance from Source (km), n (%)**				
< 67	7 (25)	14 (50)	7 (25)	.001
68 – 119	11 (41)	8 (30)	8 (30)	
120 – 468	12 (44)	7 (26)	8 (30)	
Greater than 468 km	19 (70)	6 (21)	2 (7)	
Distance missing	8 (19)	16 (37)	19 (44)	
**Wave Height (m), n (%)**				
< 6.65	31 (53)	18 (31)	10 (17)	.016
> 6.65	19 (32)	20 (34)	20 (34)	
Missing wave height	7 (21)	13 (38)	14 (41)	
**Per capita GDP (USD), mean (SD)**	29368 (2605)	20561 (3104)	12584 (2364)	<.001
**Earthquake Magnitude, mean (SD)**	8.12 (0.11)	8.06 (0.14)	8.18 (0.12)	.784
**GINI Coefficient, mean (SD)**	40.28 (0.77)	37.72 (1.12)	38.82 (1.27)	.209

*Injury. *Injury reports were only available in 22 (23.4%) events for a total of 48,462 injuries (range 45,466-51,457). A typical tsunami caused 218 injuries (median value) but the distribution was skewed by rare large-scale events (mean injured=2,339, range=0-23,176). To more accurately estimate the total number of injuries due to tsunamis, it was presumed that injuries would occur in events where deaths were reported. There were 87 tsunami events with fatalities; when the median and mean for injuries were applied to the remaining 65 events with fatalities, it was estimated that between 14,170 and 152,035 unreported tsunami-related injuries occurred worldwide between 1900 and 2009.

***Systematic Literature Review***

*Mortality.* Of the 33 articles reviewed, 12 reported on mortality, including mortality counts or rates and/or risk factors for death (Table 4). Age and sex were described as risk factors for death in three articles, all from the 2004 Indian Ocean tsunami 28 ^,^ 32 ^,^ 37. In both Indonesia and Sri Lanka, significantly higher death rates were reported among females who were 1.4 to 2.1 times more likely to perish than males. Significantly elevated risk of death was also observed in children (1.8 to 4.3 times) and older adults (2.1 to 3.1 times) who were more likely to die compared with younger/middle age adults. Other risk factors included education which was inversely associated with mortality risk 28; fisheries-based livelihoods 28 ^,^ 32indoor location at the time of the tsunami and home destruction 28 and the physical environment 15. The majority of tsunami deaths were due to drowning 11 ^,^ 14.

**Table 4: Primary research articles describing tsunami related deaths and individual risk factors (N=12):** 

Article	Country	Mortality		Sex as a risk factor	Age as a risk
		N	Rate		
Tsuji et al., 1995^15^	Indonesia	223	6.9% (Lampon); 3.9% (Rajekwesi & Pancer)	Not reported	Not reported
Tsuji et al., 1995^16^	Flores Island	1,690	Not reported	Not reported	Not reported
Davies et al, 2003^17^	Papua New Guinea	1,600	Not reported	Not reported	Not reported
Brennan, 2005^18^	Indonesia	~5,460-6,090	70% (Calang)	Not reported	Not reported
Johnson et al, 2005^20^	Thailand	25	Not reported	Not reported	Not reported
Nishikiori et al., 2006^28^	Sri Lanka	457	12.6%	Higher mortality was observed among females at 17.5% vs. 8.2% for males.	Elevated mortality among children (<5yrs: 31.8% and 5-9 yrs: 23.7%) and the elderly (15.3%) as compared to 7.4% for adults 20-29 yrs.
Nishikiori et al., 2006^29^	Sri Lanka	446	0.25 deaths / 10,000 population	Not reported	Not reported
Rodriguez et al., 2006^31^	India and Sri Lanka	250,000	Not reported	Not reported	Not reported
Rofi, et al., 2006^32^	Indonesia	295	13.9% (CI:12.4-15.4)	Risk of mortality was 1.9 (CI:1.5-3.0) times greater in females than males.	Elevated risk of death for children <10yrs (2.3, CI: 1.6-3.4) and adults >60yrs (3.1, CI: 1.9-4.9) as compared to 20-39 yr olds.
Roy, 2006^33^	India	62	0.85 deaths / 10,000 population	Not reported	Not reported
Doocy et al, 2007^36^	Indonesia	131,066	Modeled rates of 23.7% (exposed population)	Not reported	Not reported
Doocy et al, 2007^37^	Indonesia	1,642	16.3% (crude) and 14.1% (adjusted)	Higher mortality rate in females (16.4%) than males (12.0%). Risk for death was 1.4 (CI: 1.3-1.6) times greater in females.	Elevated mortality among children (<0-yrs: 19.8%) and older adults (60-69yrs: 22.6%, 70+yrs: 28.1%) (15.3%) as compared to 10.8% among 20-39yr olds.

*Injury.* Tsunami-related injuries were reported in 18 articles though only ten provided detailed information on injury types or risk factors (Table 5). Only one study estimated a population-based injury rate of 8.5% in Indonesia following the 2004 tsunami 42. Other articles described the proportion of care seekers with tsunami-related injuries or included only patients with tsunami-related injuries, preventing comparisons in injury patterns across events and locations. Only two studies described risk factors for tsunami-related injury, both with similar findings, in the 2004 Asian tsunami. The first, a study of patients seen by a medical team in Sri Lanka, observed that males and adults (15-64 years) seeking care for injuries were over-represented, suggesting these population sub-groups had higher injury rates 26. The second used household survey data from Indonesia and reported lower injury risk among females (OR=0.81) and the highest injury rates among those 20-49 years of age. The risk factors for injury were opposite those of mortality suggesting that those more likely to survive the tsunami were also more likely to be injured 42. The most common types of injuries were wounds due to physical impact with debris, fractures, and near drowning and/or aspiration 21 ^,^ 22 ^,^ 23 ^,^ 25 ^,^ 26 ^,^ 30 ^,^ 42 . As compared to other types of natural disasters, tsunamis often result in relatively high mortality but have lower rates of injury 11.

**Table 5: Primary research articles describing types of tsunami related injury (N=10):** 

Article	Country Affected	Injuries Reported		Injury Type						Notes
		# injured / cases	Injury Rate	Respiratory Injury or Near Drowning	Musculo-skeletal or Orthopedic Injury	Traumatic Injury / Wound	Dermatologic Injury	Gastro-intestinal Injury	Other	
Calder et al, 2005^19^	Sri Lanka	100	Not reported							Open fractures repair, wound debridement and skeletal traction were the most common procedures
Lee et al, 2005^21^	Indonesia	1958	Not reported	27%		72%				
Lim et al, 2005^22^	Sri Lanka	4710	Not reported	28%		29%			34% of cases were non- tsunami related chronic conditions	
Maegele et al, 2005^23^	Thailand	17	Not reported		Closed fracture 35%; open fracture 24%	Soft tissue hip/ lower extremity 88%; soft tissue upper extremity 29%			Head injuries 18%, hemopnueu-mothorax 18%, thoracic trauma 14%	
Johnson et al, 2005^40^	Thailand	1357	Not reported	n=31 (2%)	n=33 (2%)	n=65 (5%)			Head, n=18 (1%); abdominal n=12 (1%), chest/ thoracic (n=3, 0%)	Retrospective study of facility data. Reports on injuries and evolution of pathology.
Chambers et al, 2006^24^	Indonesia	71	Not reported		9% fracture management	34% wound debridement			24% changing dressing under anesthetic	Reports on surgical procedures only; 69% were for tsunami-related injuries.
Fan, 2006^25^	Indonesia	1021	Not reported	32%	11% musculo-skeletal	25%	10%	10%	2% neurologic injuries	
Kwak, 2006^26^	Sri Lanka	2807	Not reported	33%	22% orthopedic	8%	13%	4%	4% headache	Adults and males had higher injury rates
Redwood –Campbell et al, 2006^30^	Indonesia	271	12% of cases were tsunami related							Most tsunami related injuries were fractures, wounds, and aspiration pneumonia.
Doocy et al, 2009^42^	Indonesia	707	8.5% (CI: 7.9-9.2)	4%	8% fractures	75%			13% other (unspecified)	Lower injury risk among females (OR=0.81, CI 0.61-0.96); highest injury rate among 20-49yr age group

## Discussion

**Main Findings**

From 1900 to 2009, approximately 2.5 million people were affected by tsunamis including over 255,000 deaths and an estimated 50,000 injuries. The mortality and population affected estimates presented in this study are consistent with other reviews of global tsunami events 11 It is likely that the number injured is underestimated, given the low frequency with which this figure was reported, particularly in the first half of the 20^th^ century. When the mean and median numbers injured were applied to events that resulted in deaths it was estimated that between 10,900 and 116,950 unreported tsunami-related injuries occurred worldwide during this time period. It is important to note, that studies have shown that that ratio of deaths to injuries following a tsunami is typically significantly higher compared with other natural disaster types 11.

This study is the first to examine the influence of place and event characteristics on tsunami mortality. Analyses of tsunami run-up data from 1900 to 2009 reveal that events occurring in the South East Asian region were significantly more likely to result in greater numbers of deaths compared to other regions, and this finding persisted even after excluding the 2004 Indian Ocean tsunami. Examination of the relationship between mortality levels and the two poverty measures (World Bank income level and per capita GDP) demonstrate that risk of mortality event is significantly higher in low-income countries. Lastly, certain event characteristics are more likely to be predictive of mortality than others. Increased wave height and closer proximity to the source were associated with higher mortality levels whereas earthquake magnitude was not associated mortality. A number of other factors that were not examined in this study have been shown to influence tsunami damage and impacts including wave velocity, water depth and submarine topography 10. Ecological assessments such as this review may mask significant in-country differences in mortality risk (e.g. population density, rural or urban location, geographic variations and individual-level socio-demographic factors).

Findings from the systematic literature review of studies examining tsunami-related mortality and injury contribute to understanding the primary causes of death and types of injury, as well as factors that place certain populations at increased risk. The most common cause of tsunami-related death was drowning, and the most frequently reported injuries included wounds and lacerations, fractures, and near drowning and/or aspiration. When reported the mortality risk was higher among females and the very young and old and injuries were more common among middle-aged men. Additional mortality risk factors included location during the event and fisheries-based livelihood. Few studies assessed or found relationships between socioeconomic status and mortality risk, although that one study found an inverse association between education level and mortality 28 which suggests that low socioeconomic status may place individuals at increase mortality risk. This suggests that preparedness efforts target specific population groups for the prevention of deaths and injuries. Future studies on both tsunami-related injury and mortality risk would benefit from incorporating additional socio-demographic measures to gain a more comprehensive understanding of risk factors.

A number of strategies could be adopted by the international community and vulnerable countries to mitigate the short and long term impacts of future tsunami events. In the 1998 Papua New Guinea tsunami factors that contributed to higher mortality during this event included concentration of populations in vulnerable areas and failure of residents in affected areas to timely evacuate. In contrast, deployment of medical assistance and international support immediately following the tsunami played a large role in preventing further loss of life 13.With respect to public health in particular, a greater focus on ongoing disease surveillance, the appropriate targeting of aid to those in most need, and strengthening of health care systems 11 can help to mitigate the medium to long term health impacts of tsunamis. Advanced tsunami warning systems may vastly improve early detection, and education campaigns can play a crucial role in improving awareness about tsunami risk and mitigation 10. Policies enacted by the Sri Lankan government following the 2004 tsunami highlight some of the challenges to longer term disaster mitigation where enactment of a buffer zone policy that forced the relocation of coastal communities had deleterious social and economic impacts 12. In adopting disaster mitigation policy, governments should consider the contribution not only of physical vulnerability (i.e. distance of communities from coastal areas) but also social vulnerability, such as livelihoods, to increased disaster risk and the potential short and long term impacts such policies may have on affected communities.

***Limitations***

Systematic reviews face numerous limitations. The effects of tsunamis are the subject of gross approximations and aggregations which result in a great deal of imprecision. The availability and quality of data has likely increased and improved over time, however, in many events deaths are unknown or unrecorded. For a significant number of events no data are reported for injured, displaced, and affected populations; this likely contributes to a substantial underestimation of the impacts of tsunamis on human populations. Inconsistencies and errors were common in the data files from the two different sources, and in some cases inclusion criteria were not ideal for the purposes of this review which created a challenge in reconciling event lists. Additionally, mainly due to the small number of tsunami run-up events reported over the study period, we were unable to perform more complicated statistical analyses that would have provided estimates of the independent effects of place and event characteristics on tsunami related death. When combined with the relatively small number of tsunami events, uncertainty in the historical record limits the conclusions that can be drawn about the impact of tsunamis on human populations. A principal limitation of the literature review is the fact that only English language publications were included; this likely contributed to incomplete coverage of studies published in other languages originating from low and middle income countries.

## Conclusions

From 1900 to 2009, a total of 250,000 tsunami-related deaths and close to 50,000 injuries, respectively, were reported, the majority of which were concentrated in the 2004 Indian Ocean tsunami. An estimated 2.5 million people were affected by tsunamis during this time period. While mortality estimates presented in this study are consistent with those reported in other studies, particularly for the 2004 Indian Ocean Tsunami, the injury figure may be an underestimate of the true value given low reporting levels. The distribution of tsunami related deaths varied greatly by region and economic development level. Findings from the historical event review indicate that the South East Asian region and poorer countries were more likely to experience higher mortality was associated with larger wave height and closer proximity to the source.

The primary cause of tsunami-related mortality was drowning and, although a number of injury types were reported following tsunamis, the ratio of dead to injured is much greater in tsunamis as compared to other natural disaster types. Risk factors for tsunami-related death included female sex and very young and old age. Tsunami losses are likely to increase in future years due to population growth in high risk seismic areas. Increased attention to tsunami prevention and mitigation strategies, with a focus on areas most prone to tsunamis and populations at greater risk is necessary. While strategies that are specific to the development level and country context are important, global initiatives such as early warning systems are essential for further tsunami risk mitigation.

## Competing Interests

The authors have declared that no competing interests exist.

## Correspondence

Shannon Doocy, Johns Hopkins Bloomberg School of Public Health, 615 N. Wolfe St, Suite E8132, Baltimore, MD 21230. Tel. 410-502-2628. Fax: 410-614-1419. Email: sdoocy@jhsph.edu.

## Appendix 1

### PRISMA Checklist

Download PDF
